# *SCN1A*-Related Epilepsies: Clinical Spectrum and Mechanisms

**DOI:** 10.3390/cimb48101023

**Published:** 2026-10-02

**Authors:** Ja-Un Moon, Tae-Hoon Eom

**Affiliations:** Department of Pediatrics, College of Medicine, The Catholic University of Korea, Seoul 06591, Republic of Korea; gonicky@catholic.ac.kr

**Keywords:** *SCN1A*, Nav1.1, GABAergic interneuron, Dravet syndrome, genetic epilepsy with febrile seizures plus (GEFS+), developmental and epileptic encephalopathy (DEE), genetic diagnosis, genotype–phenotype correlation

## Abstract

Pathogenic variants in *SCN1A* are associated with a broad and overlapping spectrum of epilepsies, ranging from febrile seizures and genetic epilepsy with febrile seizures plus (GEFS+) to severe developmental and epileptic encephalopathies (DEEs), most notably Dravet syndrome. *SCN1A* encodes the voltage-gated sodium channel α-subunit Nav1.1, which has a critical role in inhibitory interneurons. In classical loss-of-function-associated Dravet syndrome, reduced Nav1.1 function contributes to impaired inhibitory neuronal excitability and network hyperexcitability. However, *SCN1A*-related disorders are mechanistically heterogeneous, with gain-of-function or mixed-function pathogenic variants being associated with clinically distinct phenotypes, including early-onset DEE and familial hemiplegic migraine type 3 (FHM3). This narrative review integrates functional variant classification with genotype–function–phenotype relationships, clinical diagnosis, and phenotype-specific treatment considerations. Particular emphasis is placed on early molecular testing, careful variant interpretation, and the distinction between chronic sodium-channel-blocker avoidance in classical loss-of-function-associated Dravet syndrome and potential mechanism-informed treatment strategies for selected gain-of-function phenotypes. We also summarize emerging *SCN1A*-targeted approaches, including antisense oligonucleotide therapy, gene activation, and interneuron-targeted gene replacement. A clearer understanding of variant-specific mechanisms and longitudinal clinical features may improve diagnostic precision, individualized management, and the development of disease-modifying therapies for *SCN1A*-related epilepsies.

## 1. Introduction

Epilepsies caused by pathogenic variants in *SCN1A* represent one of the most intensively studied examples of monogenic seizure disorders [1,2,3,4,5,6,7]. *SCN1A* encodes the α-subunit of the voltage-gated sodium channel Nav1.1, which is broadly expressed in the central nervous system and has a critical functional role in inhibitory interneurons [8,9,10,11]. Since the initial identification of *SCN1A* variants in patients with severe myoclonic epilepsy of infancy (SMEI), later termed Dravet syndrome, the clinical spectrum associated with this gene has expanded considerably [1,2,3,4,5,6,7,12,13,14,15,16,17].

Current evidence indicates that *SCN1A*-related disorders form a broad phenotypic spectrum, ranging from febrile seizures and genetic epilepsy with febrile seizures plus (GEFS+) to intractable childhood epilepsies and severe developmental and epileptic encephalopathies (DEE), including Dravet syndrome [2,3,4,5,7,14,15,16,17]. Although many epilepsy-associated *SCN1A* variants are associated with loss of Nav1.1 function, gain-of-function or mixed-function variants have also been linked to clinically distinct phenotypes, including familial hemiplegic migraine type 3 (FHM3) and early-onset DEE [18]. Even within a single family, considerable intrafamilial variability can be observed, highlighting the challenge of establishing robust genotype–phenotype correlations in *SCN1A*-related disorders [2,4,5,7,14,15,16,17].

At the mechanistic level, loss of Nav1.1 function and impaired inhibitory interneuron excitability provide an important framework for understanding Dravet syndrome and many *SCN1A*-loss-of-function-associated epilepsies [8,9,10,11,19,20,21,22]. However, the functional and network consequences of *SCN1A* variants are context-dependent and may vary according to the affected neuronal population, developmental stage, genetic background, and variant-specific biophysical effects [10,11,18,19,20,21,22]. These considerations help explain the marked phenotypic diversity across the *SCN1A*-related disorder spectrum.

This narrative review summarizes the clinical spectrum, diagnosis, pathophysiology, and therapeutic considerations of *SCN1A*-related disorders. Its specific focus is the integration of functional variant classification with genotype–function–phenotype relationships, phenotype-specific clinical decision-making, and emerging mechanism-targeted therapies. A clearer understanding of these relationships may improve diagnostic precision, support early genetic testing, and inform individualized management and future treatment development [23,24].

## 2. Literature Search Strategy

This narrative review was based on a targeted literature search of PubMed/MEDLINE, Embase, and Google Scholar for English-language publications from database inception to 31 August 2026. Search terms included “*SCN1A*,” “Nav1.1,” “Dravet syndrome,” “severe myoclonic epilepsy of infancy (SMEI),” “genetic epilepsy with febrile seizures plus (GEFS+),” “developmental and epileptic encephalopathy (DEE),” “familial hemiplegic migraine type 3 (FHM3),” “gain-of-function,” “loss-of-function,” “genotype–phenotype,” “functional assay,” “sodium-channel blocker,” “genetic testing,” “electroencephalography (EEG),” “magnetic resonance imaging (MRI),” and “precision therapy.” Reference lists of relevant reviews, consensus statements, guidelines, and primary studies were also screened to identify additional relevant publications.

We prioritized recent international consensus statements and clinical guidelines, randomized and other prospective clinical trials, large observational cohorts, genotype–phenotype and functional studies, and key preclinical studies relevant to disease mechanisms and emerging molecular therapies. Studies were selected for relevance to the clinical spectrum, diagnosis, pathophysiology, genotype–function–phenotype relationships, treatment, and long-term outcomes of *SCN1A*-related disorders. Case reports and small case series were considered when they provided information on rare phenotypes, including gain-of-function or mixed-function disorders, early-onset DEE with movement disorder or arthrogryposis, acute encephalopathy, or unusual treatment responses. Articles were excluded when they were not relevant to *SCN1A*-related disorders, lacked sufficient clinical or functional detail, duplicated higher-quality evidence, or were not available in English.

## 3. *SCN1A*-Related Epilepsy Spectrum

*SCN1A*-related epilepsies encompass a broad phenotypic spectrum, ranging from febrile seizures and GEFS+ to severe DEEs, including intractable childhood epilepsy with generalized tonic–clonic seizures (ICE-GTC), focal seizure phenotypes, myoclonic–atonic epilepsy (MAE) and Dravet syndrome [2,3,4,5,7]. The recognition of this spectrum has shifted the conceptualization of *SCN1A*-related disorders from a single prototypical syndrome to a group of overlapping phenotypes with shared genetic underpinnings but variable functional mechanisms and clinical outcomes [2,3,4,5,7].

Simple febrile seizures represent the mildest end of the *SCN1A*-related epilepsy spectrum [2,4,7,14,15,16,17]. They typically occur between 6 months and 5 years of age in association with fever and are brief, generalized seizures lasting less than 15 min, without focal features or recurrence within 24 h. Although most simple febrile seizures are not attributable to *SCN1A*, pathogenic or likely pathogenic *SCN1A* variants may be associated with febrile seizures plus (FS+), or the GEFS+ spectrum [2,4,7,14,15,16,17].

GEFS+, a core component of the *SCN1A*-related epilepsy spectrum, is a familial epilepsy syndrome characterized by febrile seizures persisting beyond the usual age range and/or afebrile seizures, including generalized tonic–clonic, myoclonic, absence, or focal seizures. In families with autosomal-dominant inheritance, pathogenic *SCN1A* variants are an important genetic cause of GEFS+, and affected relatives carrying the same variant may show marked variation in seizure type and severity [2,4,7,14,15,16,17].

Other *SCN1A*-associated epilepsies include ICE-GTC, focal seizure phenotypes, and MAE. ICE-GTC is characterized by generalized seizures, including frequent generalized tonic–clonic seizures and sometimes absence seizures, with onset in infancy or childhood; seizures are often treatment-resistant [2,7]. Focal seizures, including focal motor or autonomic seizures, may occur in *SCN1A*-related disorders and can be prominent in patients with severe or atypical phenotypes. MAE, also known as Doose syndrome, has been reported in association with *SCN1A* variants and may occur within the broader *SCN1A*-related epilepsy spectrum [2,3,7].

Dravet syndrome is a rare developmental and epileptic encephalopathy, with reported population-based incidence estimates of approximately 1 per 15,000–22,000 live births [25,26]. Population-level incidence estimates for the broader *SCN1A*-related spectrum remain less well defined than those for Dravet syndrome, reflecting marked phenotypic heterogeneity and variable penetrance [2,7].

Within this spectrum, Dravet syndrome, historically termed SMEI, occupies the severe end of the predominantly loss-of-function-associated phenotypes [2,3,5,7,13,27]. Dravet syndrome is the prototypical severe *SCN1A*-related disorder, most often associated with haploinsufficiency or marked loss of Nav1.1 function. It is most commonly associated with de novo truncating, canonical splice-site, or functionally deleterious missense pathogenic variants [2,3,6,7,10,11,12,13]. Dravet syndrome is characterized by onset in the first year of life with prolonged hemiclonic or generalized tonic–clonic seizures, often precipitated by fever or hyperthermia, followed by the emergence of multiple seizure types, including myoclonic, atypical absence, focal, and atonic seizures [2,5,7,24,27,28]. In genetically predisposed infants, fever temporally associated with vaccination may trigger an initial seizure; vaccination itself does not cause *SCN1A*-related epilepsy [24]. More than 80% of patients with clinically diagnosed Dravet syndrome harbor a pathogenic *SCN1A* variant, most commonly a de novo variant causing loss of Nav1.1 function [2,3,6,13,24].

The phenotypic spectrum also extends beyond the conventional febrile seizure–GEFS+–Dravet framework. Gain-of-function or mixed-function *SCN1A* variants have been associated with FHM3 and with clinically distinct early infantile or neonatal-onset DEE; movement disorder, dystonia, or congenital arthrogryposis may occur in selected phenotypes [18,29]. These disorders differ from classical Dravet syndrome in age at onset, variant location and functional consequence, neurodevelopmental course, and potential response to antiseizure medications (ASMs) [2,7,11,18,29].

Beyond these established syndromic labels, pathogenic *SCN1A* variants have been reported in other DEE phenotypes, including epilepsy of infancy with migrating focal seizures, infantile spasms or West syndrome, and Lennox–Gastaut syndrome [2,3,7]. Neurodevelopmental impairment, including intellectual disability and autistic features, may accompany *SCN1A*-related disorders, particularly severe DEE phenotypes [2,7]. These presentations require careful assessment of variant pathogenicity, functional consequence, and possible alternative or additional genetic diagnoses [2,7,11,30].

Even within a single family carrying the same *SCN1A* variant, phenotypes range from isolated febrile seizures or GEFS+ to more severe epilepsy phenotypes, including Dravet syndrome, emphasizing substantial intrafamilial variability and the contribution of genetic background and environmental modifiers. Across the full spectrum, genotype–phenotype associations are probabilistic rather than deterministic and may be influenced by variant location and biophysical effect, mosaicism, inheritance pattern, modifier genes, developmental stage, age at seizure onset, fever sensitivity, seizure burden, and treatment exposure [2,4,14,15,16,17,25,30]. The clinical, functional, and therapeutic characteristics of major *SCN1A*-related phenotypes are summarized in Table 1.

## 4. Diagnosis

The diagnosis of *SCN1A*-related disorders requires the integration of the clinical phenotype, seizure history, electroencephalographic findings, neuroimaging, and molecular testing [2,5,7,24]. *SCN1A*-related epilepsy should be considered in infants or young children with recurrent fever-sensitive seizures, prolonged hemiclonic or generalized tonic–clonic seizures, seizure onset in the first year of life, unexplained developmental slowing, or a family history of febrile seizures or epilepsy [2,3,4,5,7,24,27]. In particular, genetic testing should be initiated promptly in developmentally normal infants aged 2–15 months with a first prolonged hemiclonic seizure or focal or generalized convulsive status epilepticus associated with fever or following vaccination, in the absence of an alternative etiology [24]. Genotype-informed prediction models that combine *SCN1A* variant characteristics with early clinical features, including age at seizure onset, may assist in estimating the likelihood of Dravet syndrome versus milder *SCN1A*-related phenotypes; however, these tools complement rather than replace expert clinical assessment and longitudinal follow-up [39]. GEFS+ should be considered in individuals with febrile seizures persisting beyond the usual age range and/or afebrile seizures, particularly when there is a family history of febrile seizures or epilepsy with variable expression among relatives; however, GEFS+ is clinically and genetically heterogeneous and does not require identification of an *SCN1A* variant for diagnosis [2,4,7,14,15,16,17].

EEG findings evolve with age and phenotype [2,5,24,40]. In Dravet syndrome, EEG may be normal early in the disease course, whereas later recordings may demonstrate background slowing and generalized, focal, or multifocal epileptiform discharges. Photosensitivity, including photoparoxysmal responses or photically induced seizures during intermittent photic stimulation, may occur in some patients but is not required for diagnosis [2,5,24,40]. Brain MRI is typically normal at presentation in Dravet syndrome; later nonspecific abnormalities, including cerebral or cerebellar atrophy, hippocampal sclerosis, white-matter signal abnormalities, ventricular enlargement, and cortical dysplasia, may be observed in a subset of patients [2,5,24,40]. MRI is nevertheless important to exclude structural causes of focal seizures, developmental delay, or epileptic encephalopathy, particularly in patients with atypical electroclinical features [2,5,7,24].

An epilepsy multigene panel that includes *SCN1A* is an efficient first-line molecular test in patients with suspected genetic epilepsy because it allows simultaneous evaluation of phenotypically overlapping disorders and assessment of relevant differential-diagnosis genes [2,7,23,24]. The selected assay should include sequencing with adequate coverage and copy-number variant analysis to detect intragenic deletions or duplications; if panel testing is nondiagnostic or the phenotype is atypical, exome or genome sequencing, preferably with parental samples, should be considered [2,23,41]. Variant classification should follow ACMG/AMP criteria and integrate population frequency, computational and in silico data, segregation, de novo status, phenotype consistency, and functional evidence when available [42]. A variant of uncertain significance should not be used as the sole basis for diagnosis, prognosis, or mechanism-based treatment selection. Following identification of a pathogenic or likely pathogenic *SCN1A* variant, parental testing helps clarify inheritance, assess for parental mosaicism, and inform recurrence-risk counseling [2,43]. Although many Dravet-associated variants are de novo, parental mosaicism, including mosaicism with potential germline involvement, can explain recurrence in families with apparently unaffected parents [43].

Differential diagnosis should be guided by age at onset, seizure semiology, developmental trajectory, EEG, MRI, family history, and genetic findings [2,5,7,24]. Disorders that may mimic Dravet syndrome or other *SCN1A*-related epilepsies include *PCDH19*-related epilepsy and *SCN1B-*, *GABRA1*-, *STXBP1*-, *CHD2*-, *HCN1*-, *SCN2A*-, and *SCN8A*-related epilepsies [7,44]. Distinguishing features may include female-limited inheritance and seizure clustering in *PCDH19*-related epilepsy; GEFS+ or Dravet-like phenotypes associated with *SCN1B*; generalized epilepsy phenotypes, including childhood absence epilepsy or genetic generalized epilepsy, associated with *GABRA1*; earlier developmental impairment or epileptic spasms in *STXBP1*-related disorders; MAE or photosensitive generalized epilepsy associated with *CHD2*; early-onset febrile seizures, generalized epilepsy, or DEE associated with *HCN1*; and variant-specific gain-of-function versus loss-of-function mechanisms in *SCN2A* and *SCN8A* disorders, which may have different treatment implications [7,44,45,46,47,48,49]. When the clinical phenotype is inconsistent with the detected variant, its classification, or its predicted functional mechanism, clinicians should reassess the variant interpretation and consider alternative or additional genetic and structural etiologies [2,7,24,42].

## 5. Pathophysiology

**Figure 1:**
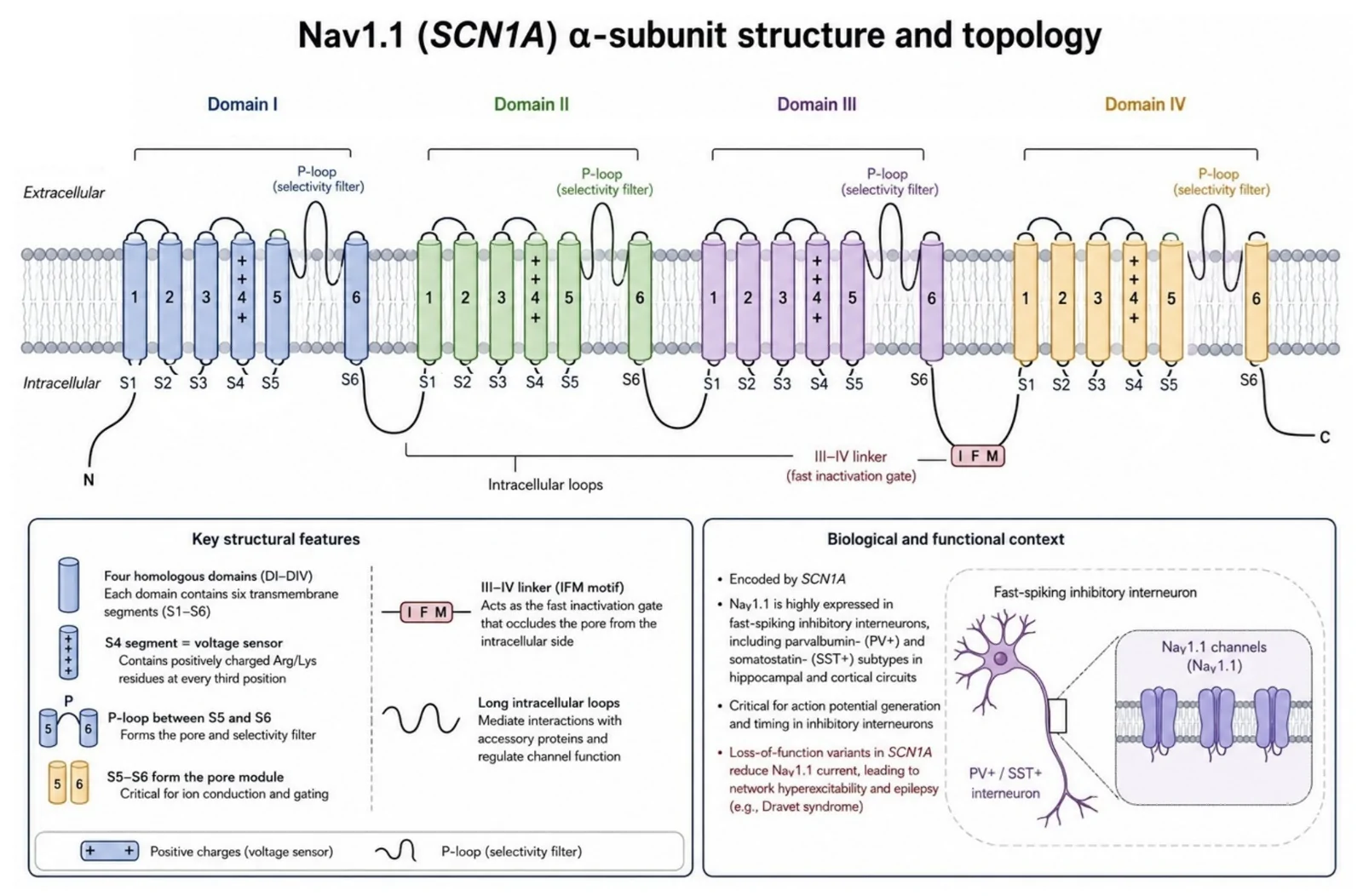
Structural topology and functional context of the Nav1.1 (*SCN1A*) voltage-gated sodium channel α-subunit. The schematic illustrates the transmembrane arrangement and key functional domains of the Nav1.1 α-subunit. The polypeptide chain comprises four homologous domains (DI–DIV), each containing six transmembrane segments (S1–S6). The S4 segments serve as the voltage sensors, characterized by positively charged amino acid residues. The pore loops between S5 and S6 form the ion-conducting pore and contain the selectivity filter. The intracellular linker between DIII and DIV contains the hydrophobic IFM (isoleucine-phenylalanine-methionine) motif, which is essential to rapid inactivation. Source: Created by the authors.

**Figure 2:**
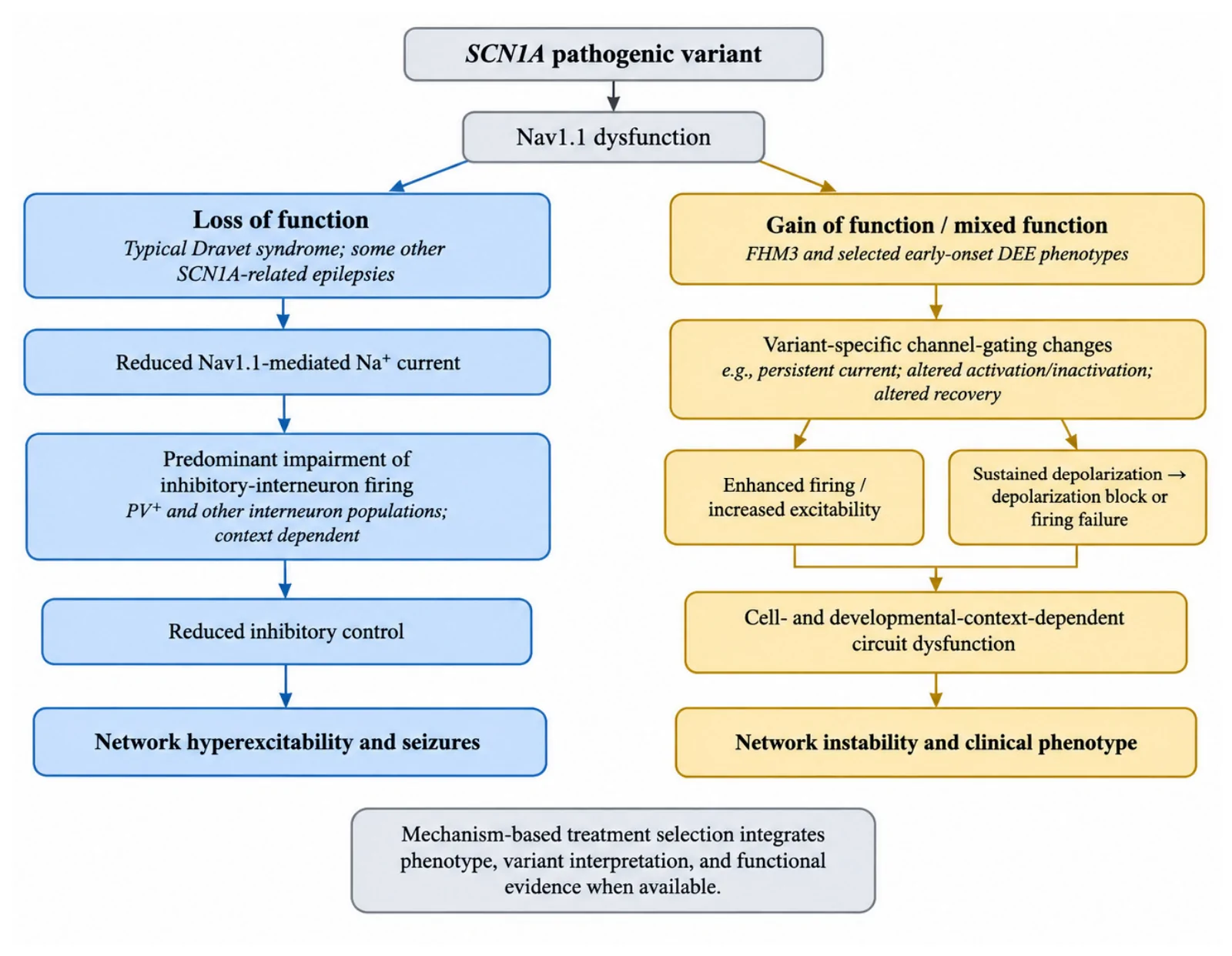
Context-dependent mechanisms of *SCN1A*/Nav1.1 dysfunction. Loss-of-function variants, typical of Dravet syndrome, predominantly impair inhibitory interneuron function and reduce inhibitory control. Gain-of-function or mixed-function variants can produce variant-specific gating abnormalities with divergent effects on firing and circuit function, including increased excitability or depolarization block. Thus, clinical phenotype and mechanism-based treatment implications depend on variant function, cellular context, and developmental stage. Abbreviations: DEE, developmental and epileptic encephalopathy; FHM3, familial hemiplegic migraine type 3; PV, parvalbumin; *SCN1A*, sodium voltage-gated channel alpha subunit 1. Source: Created by the authors.

*SCN1A* encodes the Nav1.1 α-subunit, a voltage-gated sodium channel with a critical functional role in inhibitory interneurons, particularly parvalbumin- positive interneurons, within hippocampal and cortical circuits (Figure 1) [8,10,11]. In classical Dravet syndrome and many loss-of-function-associated *SCN1A*-related epilepsies, pathogenic variants reduce Nav1.1 function and, in experimental models, predominantly impair sodium-current availability and action-potential generation in GABAergic interneurons while relatively sparing excitatory pyramidal neurons [9,10,11,19,21]. The resulting reduction in inhibitory control contributes to an excitation-inhibition imbalance and network hyperexcitability, providing a central mechanistic framework for seizure susceptibility in these disorders (Figure 2) [8,9,10,11,19,21].

Constitutive and conditional *SCN1A* mouse models provide strong support for this interneuron-centered framework [9,19,20,21]. Constitutive *SCN1A* haploinsufficiency reproduces major Dravet-like phenotypes, including spontaneous and hyperthermia-provoked seizures, motor abnormalities, and premature death [9,19]. Conditional *SCN1A* deletion in inhibitory interneurons is sufficient to produce severe epilepsy and early mortality, supporting a causal contribution of impaired inhibitory circuitry [19]. Electrophysiological recordings in these models demonstrate reduced interneuron firing and sodium current, as well as impaired action-potential initiation, with consequent network hyperexcitability [9,19,21].

Human patient-derived neuronal models provide complementary evidence for *SCN1A*-related network dysfunction [22]. However, experimental findings can differ according to the specific variant, neuronal subtype and regional identity, developmental maturity, cellular composition, differentiation protocol, and genetic background. Thus, animal and induced pluripotent stem cell (iPSC)-derived neuronal models should be interpreted as complementary and context-dependent rather than as demonstrating a single uniform cellular mechanism for every *SCN1A* variant or clinical phenotype [10,11,18,19,20,21,22,29].

The pathophysiology of *SCN1A*-related disorders therefore extends beyond a static reduction in inhibition [10,11]. Nav1.1 dysfunction may affect interneuron maturation, connectivity, and circuit development, while the impact of a given variant depends on its molecular effect, affected neuronal population, developmental stage, and genetic context [10,11,19,20,21,22]. In contrast to the canonical loss-of-function mechanism of Dravet syndrome, gain-of-function or mixed-function *SCN1A* variants are associated with clinically distinct phenotypes, including FHM3 and early-onset DEE. Their functional consequences are variant- and cell-context-dependent and may include changes in persistent current, activation or inactivation, recovery from inactivation, firing behavior, or, in some settings, depolarization block and circuit dysfunction [18,29,31]. Both loss- and gain-of-function mechanisms can therefore perturb network stability through different cellular routes and developmental windows (Figure 2) [12,13,18,29,31].

Genotype–function–phenotype relations are informative but probabilistic. Truncating and canonical splice-site variants are frequently associated with Dravet syndrome and other severe phenotypes, whereas some missense variants with less severe or distinct functional effects occur in milder phenotypes, including GEFS+; nevertheless, missense variants can also cause severe disease [2,3,5,6,7,13,30]. Marked intrafamilial variability and overlapping phenotypes among carriers of the same *SCN1A* variant indicate that the variant class cannot be the only determinant of prognosis. Variant location and biophysical effect, inheritance and mosaicism, genetic modifiers, developmental stage, age at seizure onset, and environmental exposures all may influence network vulnerability and clinical course [2,4,7,14,15,16,17,30].

These mechanistic distinctions have direct therapeutic implications, but treatment recommendations should not be generalized across the entire *SCN1A* spectrum. In classical loss-of-function-associated Dravet syndrome, chronic sodium-channel-blocker therapy may worsen seizures, so such agents are generally avoided [2,5,10,24]. By contrast, sodium-channel blockers may be beneficial in selected gain-of-function *SCN1A* phenotypes. Accordingly, mechanism-based treatment selection should integrate the clinical phenotype, variant interpretation, and functional evidence when available, rather than relying on the presence of an *SCN1A* variant alone [18,29,31]. This distinction is especially important when considering long-term therapy; evidence and practice regarding the acute treatment of convulsive status epilepticus should be considered separately from chronic sodium-channel-blocker exposure [24,32].

Mechanism-directed therapies seek to restore or augment *SCN1A*/Nav1.1 function in relevant inhibitory circuits. In preclinical Dravet models, antisense oligonucleotide–mediated *SCN1A* upregulation, catalytically dead Cas9 (dCas9)-based gene activation, and interneuron-targeted AAV gene replacement have improved inhibitory-neuron function and reduced seizure-related phenotypes [50,51,52,53]. These studies provide biological rationale for disease-modifying approaches, but preclinical findings should not be equated with established clinical efficacy. Clinical evidence, ongoing trials, and safety considerations for molecular therapies are addressed in the treatment section.

## 6. Clinical Features

Clinical manifestations of *SCN1A*-related epilepsies evolve over time and are particularly well characterized in Dravet syndrome. Beyond the phenotype classification described in Section 3, developmental and cognitive function, motor ability, behavioral and sleep comorbidities, autonomic symptoms, and risks related to prolonged seizures and intercurrent illness should be assessed longitudinally [2,7,24,28,33,49,54].

A major clinical issue in *SCN1A*-related epilepsies is the frequent presence of developmental delay and cognitive impairment, especially in patients with phenotypes on the severe end of the spectrum [2,5,7,23,28,33]. Development is often normal or near-normal before seizure onset, but many children subsequently show a developmental plateau or regression during infancy or early childhood as seizures become more frequent and prolonged [2,27,28,40]. Longitudinal studies in Dravet syndrome indicate that developmental delays usually emerge within the first two to three years of life and may involve multiple domains, including cognitive, language, motor, and behavioral development [7,23,28,33]. Cognitive outcomes vary widely across the *SCN1A* spectrum, ranging from normal or mildly affected intellectual function in some FS+/GEFS+ and intermediate phenotypes to moderate-to-severe intellectual disability in Dravet syndrome and other DEEs [2,4,7,23,28]. Intellectual outcomes are heterogeneous and should not be inferred from seizure burden or variant class alone [2,7,28,30].

Motor abnormalities commonly accompany the epileptic and cognitive phenotype in *SCN1A*-related disorders [7,23,28,33]. Gait ataxia, clumsiness, hypotonia, and poor coordination are frequently observed in Dravet syndrome, and some individuals later develop more complex motor impairments, including crouch gait and progressive ambulatory difficulty [5,24,28,32]. Motor problems may progress over time and contribute substantially to functional disability in adolescence and adulthood; contributions from the underlying disorder, recurrent seizures, treatment effects, and reduced physical activity should be considered in multidisciplinary assessment [24,28,32,33].

Behavioral difficulties are also frequent and can include hyperactivity, impulsivity, inattention, emotional dysregulation, anxiety, and autistic features [7,33]. Such symptoms are especially prominent in severe *SCN1A*-related disorders and can substantially affect family burden, school adaptation, and long-term quality of life [24,33]. Sleep disturbances are common and may exacerbate daytime behavioral difficulties and caregiver burden. Autonomic dysregulation is also increasingly recognized as part of the broader clinical burden, although its expression and mechanisms vary among individuals [2,7,24,32,33].

Acute encephalopathy is an important and potentially catastrophic complication of Dravet syndrome. It may occur after prolonged status epilepticus during febrile or infectious illnesses and can be associated with brain edema, severe impairment of consciousness or persistent coma, death, and severe neurological sequelae [55]. In a Japanese mortality survey, sudden death and acute encephalopathy with status epilepticus were among the principal causes of death in Dravet syndrome [56]. Acute encephalopathy may also occur in children without classical Dravet syndrome who carry missense *SCN1A* pathogenic variants, supporting a possible susceptibility role beyond the classical Dravet phenotype [57]. This association should be interpreted cautiously and does not establish that all acute encephalopathies in an individual with an *SCN1A* variant are mechanistically equivalent to Dravet syndrome.

Dravet syndrome is associated with substantial premature mortality, including sudden unexpected death in epilepsy (SUDEP), status epilepticus, acute encephalopathy, drowning, and other seizure-related complications [54,56]. Prognoses across the wider *SCN1A*-related spectrum remain heterogeneous: some individuals experience febrile seizures with little long-term developmental impact, whereas others develop persistent drug-resistant epilepsy, intellectual disability, and severe neurobehavioral impairment [2,7,28,30]. This variability reflects the interaction of variant-specific functional effects, inheritance and mosaicism, genetic modifiers, developmental stage, seizure burden, and environmental factors; consequently, clinical outcomes cannot be inferred reliably from genotype alone [2,6,12,13,28,30]. As increasing numbers of individuals with Dravet syndrome survive into adulthood, care should also address transition to adult services, persistent seizure and safety needs, cognitive and communication support, mobility and orthopedic issues, behavioral and psychiatric symptoms, sleep and autonomic concerns, social participation, and caregiver support [24,32,33].

## 7. Treatment

Current treatments for *SCN1A*-related epilepsies remain largely symptomatic and focus on seizure reduction, the prompt treatment and prevention of prolonged seizures, and optimization of development and quality of life. Dravet syndrome is the best-studied condition within this spectrum; accordingly, the strongest treatment evidence and most syndrome-specific recommendations apply to Dravet syndrome rather than to all *SCN1A*-related epilepsies. Treatment for the other *SCN1A*-related phenotypes should be individualized according to seizure type, electroclinical syndrome, developmental profile, comorbidities, variant mechanism when known, and the observed treatment response [7,18,24,29,34]. A Dravet syndrome-focused summary of foundational, adjunctive, rescue, and mechanism-dependent treatment considerations is provided in Table 2.

In Dravet syndrome, broad-spectrum ASMs that enhance GABAergic transmission or have mixed mechanisms are commonly used as foundational and adjunctive therapies. Valproate is widely recommended as a foundational broad-spectrum ASM, and clobazam is frequently used as an early add-on therapy; selection, dose adjustment, and sequencing should be individualized according to seizure type, tolerability, drug interactions, and local prescribing practice [24,34]. Although these agents are also used in other *SCN1A*-related epilepsies, their selection should not be based solely on extrapolation from Dravet syndrome evidence [7].

Stiripentol, typically used in combination with valproate and clobazam, has been shown to reduce convulsive seizure frequency in Dravet syndrome in randomized and observational studies and is licensed as an adjunctive therapy in many regions [24,35]. Topiramate and levetiracetam are commonly used additional agents and provide incremental benefit in some individuals, although response is variable, with complete seizure freedom remaining uncommon [24,34]. Ketogenic and modified Atkins diets are important nonpharmacologic options for patients with ongoing drug-resistant seizures and may provide additional benefit in seizure control and, in some individuals, alertness and behavior [24,34,36]. Vagus nerve stimulation is generally considered a later-line option after an inadequate response to pharmacologic treatment and usually provides modest seizure reduction rather than seizure freedom [24,34].

In classical loss-of-function-associated Dravet syndrome, chronic maintenance treatment with sodium-channel blockers—including carbamazepine, oxcarbazepine, lamotrigine, and phenytoin—may exacerbate seizures and is generally avoided [5,7,24]. This recommendation should not be generalized to all *SCN1A*-related disorders, because selected gain-of-function or mixed-function phenotypes may respond to sodium-channel blockers [18,29,31]. The acute treatment of convulsive status epilepticus is distinct from chronic maintenance therapy. Intravenous phenytoin or fosphenytoin may be considered in selected refractory circumstances under specialist and protocol-based care, but recommendations vary, and this approach should not be interpreted as universal practice in Dravet syndrome [24,32].

Several ASMs have been specifically evaluated for Dravet syndrome. Stiripentol is an established adjunctive treatment in combination with valproate and clobazam [24,35]. Fenfluramine, a serotonergic agent repurposed at a low dose for epilepsy, has produced substantial reductions in monthly convulsive seizure frequency, and in some regions, it is used earlier in the treatment pathway rather than only after failure of multiple therapies [24,32,37]. Common adverse effects include decreased appetite, weight loss, fatigue or somnolence, and diarrhea [24,37]. Echocardiographic monitoring is required because of historical concerns about valvular heart disease and pulmonary arterial hypertension, and concomitant serotonergic medications should be reviewed because of a potential risk of serotonin syndrome [24,37]. Cannabidiol has shown efficacy as an adjunctive therapy in Dravet syndrome and can provide further seizure reduction when used with conventional regimens; drug–drug interactions, particularly with clobazam, and hepatic enzyme increases require monitoring [24,38].

Soticlestat, a cholesterol 24-hydroxylase inhibitor that modulates glutamatergic transmission, showed activity in the phase 2 ELEKTRA study [58]. However, in the recent phase 3 SKYLINE trial in Dravet syndrome, soticlestat did not meet the primary efficacy endpoint for reduction in convulsive seizure frequency, despite numerical improvement and nominally significant findings for selected secondary outcomes [59]. Additional agents targeting GABAergic modulation, synaptic vesicle proteins, or metabolic pathways may further refine symptomatic control, although the current evidence base remains the most robust for Dravet syndrome [34,58].

Given the high risk of prolonged seizures and status epilepticus in Dravet syndrome and other severe *SCN1A*-related DEEs, prompt rescue treatment is an essential component of care. Families should have an individualized seizure action plan that includes the home administration of an appropriate benzodiazepine rescue medication, such as rectal or intranasal diazepam or buccal/intranasal midazolam, for prolonged or cluster seizures, and clear criteria for emergency evaluation [24,32]. In hospital, acute treatment should follow local status epilepticus protocols, generally beginning with benzodiazepines and escalating to second-line therapy when necessary [24,34]. Supportive measures during febrile illness such as hydration, comfort, and evaluation of the underlying illness are appropriate; however, antipyretic treatment should not be presented as a proven strategy for preventing seizures. Trigger avoidance and individualized emergency planning remain essential [24,32].

Beyond symptomatic ASM therapy, several emerging strategies aim to correct *SCN1A* haploinsufficiency or restore inhibitory interneuron function in classic loss-of-function-associated Dravet syndrome. Antisense oligonucleotide (ASO) approaches that enhance expression of the wild-type *SCN1A* allele have shown efficacy in mouse models, increasing Nav1.1 levels, improving interneuron excitability, and reducing seizure-related phenotypes [52]. Recent clinical data from zorevunersen (STK-001) have shown encouraging signals of clinical activity in patients with Dravet syndrome, supporting the continued clinical development of *SCN1A*-targeted ASO therapy [60]. CRISPR/dCas9-based gene activation can upregulate endogenous *SCN1A* expression in interneurons and has rescued thermally induced seizures and network deficits in preclinical models [34,50,51]. Interneuron-specific dual-AAV gene replacement approaches delivering functional *SCN1A* constructs have also reduced seizures and improved behavioral outcomes in *Scn1a*-haploinsufficient mouse models [53].

Although these gene-based approaches remain experimental, and long-term safety, optimal dosing, durability, immunogenicity, and off-target effects require continued evaluation, they illustrate a shift toward potentially disease-modifying therapies. Such approaches are primarily designed for *SCN1A* haploinsufficiency and should not be assumed to apply to gain-of-function or mixed-function disorders without phenotype- and mechanism-specific evidence [18,29,34,52,53,60]. Emerging *SCN1A*-targeted precision therapies, their development stages, trial identifiers, and key translational limitations are summarized in Table 3.

The optimal treatment of *SCN1A*-related epilepsies extends beyond seizure control to address neurodevelopmental, behavioral, and psychosocial needs, particularly in severe phenotypes [7,24,33]. Early molecular diagnosis can support timely syndrome-appropriate ASM selection, the avoidance of potentially aggravating maintenance therapy in classical loss-of-function-associated Dravet syndrome, and realistic counseling regarding prognosis, educational planning, and supportive interventions [23,24]. Multidisciplinary care such as neuropsychology, speech and language therapy, occupational and physical therapy, behavioral interventions, and social support is essential to optimizing long-term outcomes and caregiver well-being [24,33,34].

Given the marked phenotypic and prognostic heterogeneity of *SCN1A*-related disorders, treatment strategies should be individualized according to epilepsy severity, developmental profile, comorbidities, functional mechanism when established, and family priorities, with regular re-evaluation as new therapies and evidence emerge [7,18,24,30,34]. As precision therapies move toward clinical application, integrating genotype–function–phenotype information with longitudinal clinical data will be critical to selecting appropriate candidates, timing interventions, and balancing potential benefits against risks [7,18,30,34,52,53,60].

## 8. Future Directions

Rapid progress in genetics, neurobiology, and therapeutic development is shifting the field from symptomatic seizure control toward potentially disease-modifying strategies for selected *SCN1A*-related epilepsies. The most advanced approaches are directed toward classical loss-of-function/haploinsufficiency-associated Dravet syndrome, and ongoing clinical studies of *SCN1A*-targeted therapies, together with improved phenotyping, biomarkers, and natural-history data, may help define which patients are most likely to benefit and when intervention is most appropriate [60,61,62]. Emerging *SCN1A*-targeted precision therapies, their development stages, trial identifiers, and major translational limitations are summarized in Table 3.

ASO therapies, including zorevunersen (STK-001), and AAV-based gene-regulation approaches, including ETX101, have entered clinical development for *SCN1A*-positive Dravet syndrome. Gene-activation and gene replacement platforms remain at early stages of development [50,51,53,60,61]. Initial clinical data for zorevunersen support continued evaluation, whereas ETX101 is being assessed in an early-phase clinical study [60,61]. The central questions are no longer limited to seizure reduction: long-term safety, dose selection, durability, immunogenicity, vector distribution, and effects on cognition, behavior, motor function, and daily functioning must be defined before routine clinical use [53,60,61].

A major research priority is to determine the optimal therapeutic window. Preclinical studies suggest that early treatment could modify developmental and epileptic trajectories, but it is still uncertain whether intervention before or soon after clinical onset improves long-term outcomes in people [52,53]. Carefully designed trials and natural-history studies should therefore assess both age-dependent treatment effects and clinically meaningful multidomain outcomes, including seizure burden, cognition, language, behavior, motor function, sleep, adaptive function, and health-related quality of life [60,62]. Biomarkers that reflect target engagement, Nav1.1 restoration, network activity, and developmental trajectory may help guide dose selection and treatment timing, but require prospective validation.

Better genotype–function–phenotype correlations will be essential to mechanism-based treatment selection. Therapies intended to increase *SCN1A*/Nav1.1 expression are biologically mostly relevant to haploinsufficiency-associated disorders and should not be assumed to benefit gain-of-function or mixed-function phenotypes; in some such phenotypes, further augmentation of channel activity could theoretically be inappropriate or harmful [18,29,31]. Functional characterization, when feasible, should therefore be integrated with clinical phenotype, inheritance, longitudinal course, and other modifiers rather than relying on variant class alone [30].

As *SCN1A*-targeted biological therapies progress, equitable access, affordability, health system readiness, and long-term surveillance will become increasingly important. Early-life interventions raise particular ethical questions because of uncertainty about long-term benefit, irreversible or durable effects of some gene-based approaches, and the need for transparent family counseling and informed consent. International registries, postmarketing surveillance, data sharing, and collaboration among patients, caregivers, clinicians, researchers, industry, and advocacy organizations will be important for identifying rare adverse events, defining long-term benefit, and reducing disparities in access. Ultimately, progress will depend on demonstrating not only seizure reduction but also meaningful lifelong improvements in development, function, participation, and quality of life.

## 9. Conclusions

*SCN1A*-related epilepsies comprise a broad and overlapping neurodevelopmental epilepsy spectrum, ranging from febrile seizures and GEFS+ to severe DEEs, most notably Dravet syndrome. Their clinical diversity reflects variant-specific and context-dependent channel dysfunction, together with modifying genetic, developmental, and environmental factors; thus, genotype alone does not reliably determine prognosis or treatment response [2,7,30].

Determining the phenotype and functional mechanism can support early genetic diagnosis, syndrome-appropriate ASM selection, and individualized multidisciplinary care that addresses seizures as well as cognitive, behavioral, motor, sleep, and psychosocial comorbidities. In classical loss-of-function-associated Dravet syndrome, chronic maintenance sodium-channel blockers are generally avoided, whereas selected gain-of-function or mixed-function phenotypes may require different, mechanism-informed therapeutic approaches [7,18,24,29,31].

Emerging *SCN1A*-targeted approaches such as ASO therapy, gene activation, and interneuron-targeted gene replacement offer the prospect of disease modification, particularly for haploinsufficiency-associated Dravet syndrome. Their long-term safety, durability, developmental benefit, and optimal timing remain to be established, and they should not be assumed to apply to gain-of-function or mixed-function disorders without phenotype- and mechanism-specific evidence [18,29,34,50,51,52,53,60]. Continued collaboration among patients, caregivers, clinicians, researchers, industry, and advocacy organizations will be essential to translating these advances into meaningful, lifelong improvements in health, function, participation, and quality of life.

## Figures and Tables

**Table 1:** Major *SCN1A*-related phenotypes: variant mechanisms, clinical features, and treatment implications.

Phenotype	Variant Mechanism	Onset and Seizure Type	EEG	Inheritance and Development	Treatment Implications
Simple febrile seizures	Most cases are not attributable to *SCN1A*; when present, *SCN1A* effect is usually mild or incompletely characterized	6 months–5 years; brief generalized febrile seizures without focal features or recurrence within 24 h	Usually normal	Sporadic or familial; development usually normal	Usually no maintenance ASM; assess recurrent, prolonged, focal, or familial events individually
FS+/GEFS+	Often mild/partial LOF or incompletely characterized effect; genetically heterogeneous	Early childhood; febrile seizures beyond usual age range and/or afebrile generalized or focal seizures	Normal or nonspecific; generalized or focal epileptiform discharges may occur	Often autosomal-dominant with reduced penetrance and variable expressivity; development often preserved but variable	Individualize by seizure type and clinical course; do not automatically apply Dravet-specific sodium-channel-blocker avoidance
ICE-GTC, focal seizure phenotype, and MAE	Variable; partial LOF, GOF/mixed-function, or uncharacterized; MAE is genetically heterogeneous	Infancy or childhood; frequent generalized tonic–clonic, focal, and/or myoclonic–atonic seizures	Generalized, focal, multifocal, or mixed epileptiform abnormalities may occur	Sporadic or familial; developmental outcome variable	Confirm electroclinical syndrome and consider alternative/additional etiologies; tailor ASMs to phenotype and response
Dravet syndrome	Predominantly severe LOF/haploinsufficiency; truncating, canonical splice-site, and severe missense variants	First year, typically 2–15 months; prolonged hemiclonic or generalized tonic–clonic seizures and, later, multiple seizure types; fever/hyperthermia sensitivity	Often normal at early stage; later background slowing and generalized, focal, or multifocal epileptiform abnormalities	Usually de novo autosomal-dominant; parental mosaicism/inheritance uncommon. Developmental plateau/regression and cognitive, motor, behavioral, and sleep comorbidities common	Dravet-specific evidence: valproate/clobazam foundational; stiripentol, fenfluramine, and cannabidiol as adjunctive options. Chronic sodium-channel blockers generally avoided; individualized rescue plan
Early infantile/neonatal GOF or mixed-function DEE	GOF or mixed-function, variant-specific channel dysfunction	Neonatal period or early infancy, often before classical Dravet onset; early drug-resistant seizures. Movement disorder, dystonia, or arthrogryposis may occur	Variable; early multifocal epileptiform activity and/or encephalopathic background may occur	Usually de novo; developmental impairment often severe but variant-dependent	Do not assume Dravet-like treatment response. Selected GOF phenotypes may respond to sodium-channel blockers; use functional evidence and close specialist monitoring
FHM3	Predominantly GOF	Variable, commonly from childhood onward; hemiplegic migraine attacks, with seizures in a subset	Usually normal/nondiagnostic unless epilepsy coexists	Often autosomal-dominant; development often preserved in isolated FHM3	Phenotype-specific migraine care; manage coexisting epilepsy individually. Do not infer Dravet-specific ASM restrictions solely from *SCN1A* involvement
Other DEE/neurodevelopmental phenotypes	Variable: LOF, GOF/mixed-function, or uncharacterized	Usually infancy/early childhood; may include infantile spasms/West syndrome, EIMFS-like phenotype, LGS phenotype, or developmental impairment with/without epilepsy	Syndrome-specific, focal, multifocal, generalized, or nonspecific abnormalities	Usually de novo in severe DEE; development variable, often impaired in DEE	Re-evaluate phenotype, variant mechanism, and alternative/additional genetic or structural diagnoses; use syndrome-specific treatment and avoid extrapolating Dravet evidence

Note: Mechanism–phenotype relationships are probabilistic rather than deterministic. Functional evidence, phenotype, inheritance and mosaicism, age at seizure onset, EEG, longitudinal developmental course, and treatment response should be considered together. Dravet-specific treatment evidence should not be generalized to all *SCN1A*-related phenotypes [2,3,4,5,6,7,10,11,12,13,14,15,16,17,18,24,27,28,29,30,31,32,33,34,35,36,37,38]. Abbreviations: ASM, antiseizure medication; DEE, developmental and epileptic encephalopathy; EIMFS, epilepsy of infancy with migrating focal seizures; FHM3, familial hemiplegic migraine type 3; FS+, febrile seizures plus; GEFS+, genetic epilepsy with febrile seizures plus; GOF, gain-of-function; ICE-GTC, intractable childhood epilepsy with generalized tonic–clonic seizures; LGS, Lennox–Gastaut syndrome; LOF, loss-of-function; MAE, myoclonic–atonic epilepsy.

**Table 2:** Treatment considerations for Dravet syndrome and other *SCN1A*-related epilepsies.

Treatment Category	Representative Options	Main Implication
Foundational and adjunctive treatment for Dravet syndrome	Valproate, clobazam, stiripentol, fenfluramine, and cannabidiol	The strongest evidence is Dravet syndrome-specific. Selection should be made based on efficacy, tolerability, drug interactions, and regional availability.
Additional pharmacologic and nonpharmacologic options	Topiramate, levetiracetam, ketogenic or modified Atkins diet, and vagus nerve stimulation	To be considered when seizures remain drug-resistant; response is variable and complete seizure freedom is uncommon.
Chronic treatment generally avoided in classical LOF-associated Dravet syndrome	Carbamazepine, oxcarbazepine, lamotrigine, and phenytoin	Chronic sodium-channel-blocker exposure may worsen seizures. This does not automatically apply to non-Dravet *SCN1A*-related disorders.
Mechanism-dependent consideration	Sodium-channel blockers in selected GOF/mixed-function phenotypes	May be beneficial in selected patients; to be selected based on integrated consideration of phenotype, variant interpretation, functional evidence when available, and specialist oversight.
Rescue and emergency management	Diazepam or midazolam rescue; local status-epilepticus protocol	Individualized seizure action plan ought to be used. Acute management is distinct from chronic maintenance therapy; IV phenytoin/fosphenytoin may be considered only in selected refractory situations under specialist/protocol-based care.

Note: Treatment evidence is the strongest for Dravet syndrome. Chronic sodium-channel-blocker avoidance primarily applies to classical loss-of-function-associated Dravet syndrome and should not be generalized to all *SCN1A*-related phenotypes. For fenfluramine, appetite and weight, somnolence, and diarrhea should be monitored; echocardiographic surveillance should be performed; and concomitant serotonergic medications should be reviewed [5,7,18,24,29,31,32,34,35,36,37,38]. Abbreviations: GOF, gain-of-function; IV, intravenous; LOF, loss-of-function.

**Table 3:** Emerging precision therapies for *SCN1A* haploinsufficiency–associated Dravet Syndrome.

Approach	Therapeutic Rationale	Development Stage/ Trial Identifier	Key Limitation
ASO-mediated *SCN1A* upregulation: zorevunersen (STK-001)	Enhances productive expression from the non-variant *SCN1A* allele to increase Nav1.1	Clinical development; phase 3 EMPEROR, NCT06872125	Long-term safety, optimal dose, durability, and disease-modifying benefit remain under study.
AAV-mediated interneuron-targeted gene therapy: ETX101	Restores *SCN1A* expression in inhibitory interneurons	Phase 1/2 ENDEAVOR, NCT05419492	Vector distribution, immunogenicity, irreversible exposure, durability, and long-term safety require assessment.
CRISPR/dCas9 transcriptional activation	Upregulates endogenous *Scn1a* expression in inhibitory neurons	Preclinical; no clinical trial	Delivery, specificity, durability, and off-target transcriptional effects remain unresolved.
Interneuron-targeted *Scn1a* replacement	Restores functional *Scn1a* in inhibitory interneurons	Preclinical; no clinical trial	Packaging, cell specificity, dosing, immunogenicity, and translation from animal models remain challenges.

Note: These approaches are primarily designed for classical *SCN1A* loss-of-function/haploinsufficiency-associated Dravet syndrome. They should not be assumed to benefit gain-of-function or mixed-function *SCN1A* disorders without mechanism-specific evidence [18,29,34,50,51,52,53,60,61]. Abbreviations: ASO, antisense oligonucleotide; dCas9, catalytically dead CRISPR-associated protein 9.

## Data Availability

No new data were created or analyzed in this study. Data sharing is not applicable to this article.
